# Review Department

**Published:** 1890-12

**Authors:** 


### REVIEW DEPARTMENT.

LATIN GRAMMAR OF PHARMACY AND MEDICINE. By D. H. Robinson, Ph.D., Professor of Latin Language and Literature, University of Kansas; with introduction by L. E. Sayre, Ph.G., Professor of Pharmacy in, and Dean of, Department of Pharmacy, University of Kansas. Philadelphia: P. Blakiston, Son & Co. 1890.

The name of Dr. Robinson's Latin Grammar of Pharmacy and Medicine indicates the scope of the work, which has been most attractively carried out. To those who have already studied Latin, the book is an interesting bit of reading, recalling many details of grammar which have been forgotten, and supplying many technicalities of phrase and information of words which are frequently wanted for the writing of prescriptions. For the student who has been unfortunate enough to commence the study of medicine without a knowledge of Latin, the book offers a most attractive form of grammar with which to commence the study of the language, as while learning the grammar, he is constantly finding the words which he hears daily in his other lessons. We recommend the book as a standard one for the libraries of veterinarians, and trust that the authors may shortly give us another work comprehending other branches of medicine, and more extensive in its scope.

QUIZ-COMPEND, Number I. A Compend of Human Anatomy. By Samuel O. L. Potter, A.M., M.D. Fifth edition, revised and enlarged. Philadelphia: P. Blakiston, Son & Co. 1890.

As compends go, this is a very useful little book; it is especially valuable for its tables of nerves, which are classified and illustrated in a manner which is very clear for review and convenient for memorizing.

MEDICAL DIAGNOSIS. By J. M. DaCosta, M.D., LL.D. Philadelphia: J B. Lippincott Co. 1890. Seventh edition, revised.

The rearrangement and condensing of some of the chapters by Dr. DaCosta has not increased the book in size, but a number of wood cuts have been added. The publication of Dr. DaCosta's book in a German and Russian translation, and its prospective translation into French, add proof of the value of the work. While we lack a book of this sort in veterinary medicine, the principles of human diagnosis form a substitute, and can be read with profit by all students of veterinary medicine.

